# Editorial: Immunoglobulin Therapy in the 21st Century – the Dark Side of the Moon

**DOI:** 10.3389/fimmu.2015.00436

**Published:** 2015-08-26

**Authors:** Albert Farrugia, Marcella Visentini, Isabella Quinti

**Affiliations:** ^1^School of Sugery, University of Western Australia, Perth, WA, Australia; ^2^Department of Molecular Medicine, Sapienza University of Rome, Rome, Italy

**Keywords:** immunoglobulin G, immunedeficiency, dosage, safety, individualized medicine

Since its widespread introduction in the early 1980s, immunoglobulin therapy (IG) has been extensively investigated to understand its mechanism of action and clinical benefits. Research has also continued to improve its production. The survival benefits of IG for patients with primary immunodeficiency (PID) are accepted (1). The basic principle of replacing the missing protein has led to proposals for progressive increases in dosage, delivered intravenously (2), or subcutaneously (3). It is suggested that continuously increasing IG trough levels decreases pulmonary infections and damage. In contrast, other studies on large patient cohorts found no correlation between IG trough level and the incidence of pneumonia and serious infections when trough levels were raised above 400 mg/dl (4, 5). Dosage and other aspects of the therapeutic regimen remain open questions even in the mainstream indication of substitution therapy in PID. These issues have influenced the development of product modifications such as highly concentrated solutions and fast infusion rates. They have also contributed to the increased usage of 16–20% IG infused subcutaneously.

Simple replacement of the antibody defect in PID is now known to be an incomplete explanation of the mechanism of IG. A range of immunomodulatory and anti-inflammatory mechanisms are involved (6). These mechanisms are important in the role of IG in autoimmune disorders, particularly neuropathies including chronic inflammatory demyelinating polyneuropathy (CIDP), Guillain-Barré syndrome (GBS), and multifocal motor neuropathy (MMN). These indications represent the largest area of IG use in the established economies. They contribute greatly to the steady increase in demand for immunoglobulins experienced in the past 20 years, despite uncertainty in mechanisms of action. The increase in adverse events, such as thrombogenicity (7) and haemolysis (8), experienced in recent years makes a better understanding of mechanism and dosage even more important. The substantial increase in the usage of expensive IG products has also influenced developments in formulation and infusion practices. Faster infusion of more concentrated solutions will decrease hospital stay and costs. The subcutaneous route is supposedly easier and more convenient to deliver in home therapy settings (9), also potentially decreasing hospital stay. Although approved by regulatory agencies, these developments have yet to be validated through the long period of clinical practice experienced with the previous range of IG products.

The increased demand for IG has also seen the rapid development of new manufacturing methods replacing the traditional Cohn fractionation system (10). This system has demonstrated decades of safety and efficacy and caution is warranted as new methods are introduced into production and clinical use.

This Research Topic of Frontiers in Immunology has been assembled by an editorial team which have experienced sufficient “dark forebodings” (11) regarding the uncertainties outlined above. They have called upon a group of international experts to assess some of these issues from their perspective. The mechanism of IG on the immune system is explored by Nagelkerke and Kuijpers (12) who describe the different Fcγ receptors variants on immune cells and the direct IG effects at the level of the activating Fcγ receptors, including the more recently described FcgRIIc. Mitrevski et al. (13) assess analogous mechanisms in the action of IG in PID, showing that IG at replacement dosages could prime B cells to an anergic, apoptotic state through the generation of an increase in CD21^low^ B cells. Matucci et al. (14) discuss the role of benefits additional to the direct substitution of deficient IG, such as the immunomodulatory and anti-inflammatory effects of IG preparations, while Paquin-Proulx and Sandberg (15) discuss the role of immune activation in the pathology of commonest PID – common variable immunodeficiency (CVID) – and its alleviation by IG therapies.

Taken together, this body of work mitigates our “dark forebodings” regarding the lack of clarity on the mechanism of action of IG. More work is also needed to optimize therapeutic practice. Kerr et al. (16) note the desirability of progressing beyond simple, mandated, weight-based dosages in PID, and the need to approach more individualized therapeutic regimens for different PID patients. Their approach is augmented by the review of Wolf et al. (17) demonstrating how the identification of impaired IG formation may differentiate patients requiring IG from those who do not. Patient data collected through long term monitoring led Lucas et al. (18) to conclude that “The goal of replacement therapy should be to improve clinical outcome and not to reach a particular IgG trough level” supporting previous work tailoring optimal IG prophylaxis regimens to clinical and immunological markers (19). Studies such as those cited should influence treatment protocols and a negate a “one size fits all” therapeutic approach as reflected in many current guidelines. Kerr et al. (16) also note how little evidence underpins the dosage regimens used in autoimmune indications. It is to be wondered that empirically driven doses developed for the treatment of idiopathic thrombocytopenic purpura have been extended into the treatment of the various autoimmune neuropathies. The importance of individualizing treatment is also discussed by Compagno et al. (20) in their review of the use of IG in the various acquired hypogammaglobulinemic diseases. We are aware of large discrepancies between and within countries in this area, leading to the need for better evidence based on individualized treatment. Overall, these particular contributions support the need to improve outcomes in PID as shown by the study of Tabolli et al. (21) who conclude that Health-Related Quality of Life in PID patients is not related to IG therapy but to more sophisticated personal and clinical preferences.

The final component of this Research Topic of Frontiers in Immunology examines the manufacture of IG therapies and its effect on the efficacy and safety of the products. Goldacker et al. (22) show that, despite the mandatory large plasma donation pool size for IG products, the content of specific, therapeutically important antibodies is still subject to geographical influence. This is very important in the context of continuing to ensure that IG therapies are relevant in the protection of PID patients in different countries. We suggest that this requires the attention of regulatory authorities. Finally, Farrugia and Quinti (23) and Späth et al. (24) discuss the history of the manufacture of IG therapies. They suggest ways whereby changes introduced by companies to optimize yield and minimize hospital related costs might alter the repertoire of specificities and the efficacy of IG. These changes may also have played a role in recently observed surges in adverse events. These observations suggest that a more cautionary approach in the usage IG therapies is warranted.

Overall, the therapeutics of diseases caused by a deficiency or an inappropriate type of antibodies continues to be an exciting and fruitful area of clinical research, and well suited to contribute to the ongoing evolution in medicine toward more individualized and patient centric paradigms. We hope that this Research Topic of Frontiers in Immunology contributes to this debate. Such a wide debate between experts is necessary to enable the new challenges in immunoglobulin usage.

## Conflict of Interest Statement

Albert Farrugia provides contractual consulting services to companies which manufacture therapeutic immunoglobulin. Marcella Visentini and Isabella Quinti have no conflict of interest to declare.
